# Marine fish peptides (collagen peptides) compound intake promotes wound healing in rats after cesarean section

**DOI:** 10.29219/fnr.v64.4247

**Published:** 2020-08-31

**Authors:** Xue Peng, Jinfeng Xu, Yuan Tian, Wenjun Liu, Bing Peng

**Affiliations:** 1 Department of Obstetrics and Gynecology, West China Second University Hospital, Sichuan University, Chengdu, China; 2 Key Laboratory of Birth Defects and Related Diseases of Women and Children (Sichuan University), Ministry of Education, Chengdu, China; 3 West China School of Medicine, Sichuan University, Chengdu, China; 4 Jiangzhong Pharmaceutical Co., Ltd., Nanchang, China

**Keywords:** cesarean section, marine fish peptides, rat uterus, uterine scar, wound healing

## Abstract

**Background:**

Wound complications are a major source of morbidity after cesarean section (CS) and contribute to increased risks in subsequent pregnancies. In the present study, we aim to investigate the wound healing potential of a kind of oligopeptide compound, mainly derived from the marine fish peptides (MFPs), in rats after CS by biomechanical, biochemical, and histological methods.

**Methods:**

Eighty-four pregnant Sprague–Dawleyrats were randomly assigned to four groups, namely the control group and 1.1, 2.2, and 4.4 mg/kg MFP groups, respectively. The MFPs or normal saline of the equal volume was intragastrically administered every morning on the second day after CS. On days 5, 10, and 15 after the surgery, seven rats from each group were randomly selected. The samples of skin wound and uterus were harvested and then used for the following experiments and analyses.

**Results:**

Using the CS rat model, this study demonstrated that in the MFP groups, the skin tensile strength, uterine bursting pressure, and hydroxyproline (Hyp) were significantly higher than those in the control group at all three time points (*P* < 0.05). The formation of collagen and smooth muscle fibers and the expression of CD34 and connective tissue growth factor at the incision site were increasingly observed in the MFP groups (*P* < 0.05).

**Conclusions:**

MFPs have a great potential to accelerate the process and quality of wound healing in rats after CS.

## Popular scientific summary

- A kind of oligopeptide compound, mainly derived from the marine fish (marine fish peptides, MFPs), has been supposed to accelerate the process and quality of wound healing in rats after CS.
- Eighty-four pregnant Sprague-Dawley rats were randomly assigned to four groups, namely the control group and 1.1, 2.2, 4.4 mg/kg MFP group, respectively.
- It indicated that medium and high doses of MFPs feeding after CS in rats could significantly increase the tension of uterine scar and decrease the risk of uterine rupture, and promote the healing of uterine incision. Its promoting effect may be mainly related to the mechanism of promoting the formation of new capillaries in uterine scar tissue, the growth, and repair of collagen fibers and smooth muscle tissues.

Cesarean section (CS) is a common surgical procedure performed worldwide, which, when undertaken for medical reasons such as antepartum hemorrhage, fetal distress, and abnormal fetal presentation, can save the lives of mothers and newborns (1). Tracking trends in CS use around the world, the report calculated overall CS rates nearly doubling in 15 years, from 12.1% of all live births in 2000 to 21.1% in 2015 (2). The CS use is strikingly increasing in all regions, and the national CS use varied from 0.6% in South Sudan to 58.1% in the Dominican Republic. Mainland China has very high national CS use too, measuring an average of 45.7% in 2012 and 41.3% in 2016 (2, 3). However, the large increase in CS use, often for non-medical indications, accounts for negative consequences in maternal and child health (4). Wound complications are surprisingly a high problem after CS and contribute to increased risks in subsequent pregnancies (5, 6). The risk of associated morbidities is progressively increasing as the number of previous cesarean deliveries increases (7, 8).

This research evolved from clinical findings and previous studies, which suggested that although 60–80% of women who attempt a trial of labor after CS is ultimately successful, the potential risk of fatal uterine rupture exceeds 1% (9). An increased uterine contractility during labor and the surgical closure technique used for the hysterotomy were thought to be the major causes of uterine rupture (10–12). However, the process of myometrial wound healing partly determines the morphologic condition, functional behavior, and risk of uterine rupture (8, 13, 14). Buhimschi et al. (13) studied uterine wound healing in mice strains with different healing capacities and demonstrated that regenerative ability varied with genetic background and was phenotypically dependent. It also provides indirect biological evidence that a human uterine scar could be a site of active remodeling, which may extend well beyond into postpartum.

Therefore, uterine wound healing is essential for a promised future pregnancy, especially if the interval is short between two cesarean deliveries or between myomectomy and pregnancy. Some approaches have been explored to promote the healing of uterine incision (15–19), involving the tissue repair strategies, the suturing techniques, and wound protection methods. Also, many other aspects, such as obesity, diabetes mellitus, anemia, may affect wound healing after CS (20–23).

Mammalian wound healing is a dynamic and systematic process, involving mediators, blood cells, extracellular matrix, and parenchymal cells. It often follows inflammation, tissue formation, and tissue remodeling (24, 25). Generally, the biological response to tissue damage in mammals includes the following two aspects: regeneration and wound repair. Regeneration means a scarless wound healing with complete replacement of normal tissue and restoration of normal architecture and function. However, wound repair involves the migration of fibroblasts to the wound site, the formation of granulation tissue, and the laying down of collagen in a disorganized mode with the formation of fibrosis and scar tissue (26). In most cases, the injured tissue in mammals is repaired rather than restored to its original structure (13).

The healing process of the uterine wound is not well defined in humans because it is difficult to obtain serial samples of the hysterotomy scar (13, 27). Few methods are available to enhance the uterine wound healing and completely restore the function of scarred uterus. Although promoting wound healing has not been studied extensively in the uterus, there is considerable evidence that incisional integrity or tensile strength in other organs can be pharmacologically enhanced (28, 29). Some biomaterials for external use, such as collagen gel and collagen dressing, have been demonstrated to have an advantage in wound healing (30–32). However, less research has been done on the effect of oral biological agents on wound healing. In the present study, we aim to determine the wound healing potential of a kind of oligopeptide compound, mainly derived from the marine fish peptides (MFPs), in rats after CS by histological, biomechanical, and biochemical methods.

## Materials and methods

### Preparation and identification of MFPs

MFPs were mainly derived from the skin of the marine fish, which were donated by the Jiangzhong Pharmaceutical Co. Ltd. (China). The ingredients include oligopeptides powder of marine fish, freshwater fish peptides or fish collagen peptide, Taihe silk chicken, whey protein hydrolysate, resistant dextrin, purified water, food additive (maltitol, citric acid, potassium citrate, sucralose), food fortifier (casein phosphopeptide, zinc gluconate), and flavor. For more composition and preparation information, please find in Supplementary materials. MFPs are food-derived peptides that do not contain any pharmaceutical ingredients. The oral safety and quality control of MFPs conform to the Food Safety Control Standard of Jiangxi Province, China (Q/JZYY 0050S-2017).

### CS animal model

All experimental procedures involving animals were in accordance with the Guide for the Care and Use of Laboratory Animals published by the US National Institutes of Health (NIH Publication No. 85e23, revised 1985) and were approved by the Animal Ethics Review Board for Animal Studies of West China Second Hospital, Sichuan University. All animals received humane care.

Eighty-four pregnant Sprague–Dawley rats weighing between 220 and 302 g were kept under standardized laboratory conditions in an air-conditioned room (temperature approximately 21–23°C) with free access to food and water.

On day 19 of the pregnancy, the rats were anesthetized by intraperitoneal injection of 10% chloral hydrate. The CS was based on the Bowers procedure and performed under sterile conditions (29). First, a 3.0 cm longitudinal incision was made in the midline of the lower abdomen, and then a 0.5 cm longitudinal incision was made along the antimesometrial border in the midportion of each uterine horn. The rat fetuses and placentas were gently extruded through the hysterotomy. Thereafter, the uterine incision was closed by a continuous, full-thickness suture with 6-0 Coated Vicryl Plus Antibacterial Suture, (Ethicon Inc., Somerville, New Jersey, USA) and the deep abdominal fascia, as well as the peritoneum, was closed with 4-0 Suture. The skin wound was closed layer by layer using the same materials. All rats were treated with an intramuscular injection of gentamycin (14,000 units/kg) for 3 days after the surgery.

Postoperatively, the rats were randomly assigned to four groups, namely the control group and 1.1, 2.2, and 4.4 mg/kg MFP groups, respectively, with 21 rats in each group. The MFPs dissolved in saline or normal saline of the equal volume were intragastrically administered every morning on the second day after CS. On days 5, 10, and 15 after the surgery, seven rats from each group were randomly selected, anesthetized by ether inhalation, and killed by cervical dislocation. The samples of skin wound and uterus were harvested and then used for the following experiments and analyses.

### Measurement of skin wound tensile strength

The skin wound tensile strength was measured using the tonotransducer and the connected BL-402F biological function experimental system. The 1 × 0.5 cm skin strips from the midline of the abdominal wound were anchored at the tonotransducer and pulled slowly in the opposite direction until breakage. Signals of the highest tension were output and recorded.

### Measurement of uterine bursting pressure

The uterus was removed, and the left uterine horn was used to measure the bursting pressure, while the right uterine horn was used for hydroxyproline (Hyp) and histological analyses. The uterine bursting pressure was measured using the pressure transducer and the connected BL-402F biological function experimental system. First, the uterine horns were suture-ligated 1.0 cm distal to the uterine scar to prevent the leakage of perfusion fluid from the fallopian tubes. Then, the horn was opened transversely 1.0 cm proximal to the scar. A catheter was inserted into the uterine lumen and fixed in a place with a suture. The proximal end of the catheter was connected to a 50 mL injector, and a pressure transducer was connected by a three-way valve. The pressure transducer was connected to an amplifier-recorder BL-402F. Normal saline was injected into the horn slowly until the horn burst and leakage occurred. The highest pressure obtained was designated the bursting pressure (29).

### Histological study

The uterine specimens were prepared and collected from the longitudinal scar. The tissue scar was fixed in 4% paraformaldehyde for 48 h and embedded in paraffin. A 5-μm serial paraffin section was prepared and stained with hematoxylin–eosin (H&E) for histomorphological evaluation under the light microscope.

Masson’s trichrome staining was also used to evaluate the degree of collagen formation due to its ability to stain collagen to a blue color and the smooth muscle fiber to a red color. Morphological findings were semiqualitatively assessed under the light microscope using a scorning system including the percentage and intensity of positive cells. Briefly, the percentage score was defined as 1 (<30%), 2 (30–60%), and 3 (>60%). The staining intensity was defined as 0 (none), 1 (weak), 2 (moderate), and 3 (strong). The final score was calculated by multiplying the percentage score by the intensity score of positive cells (33). Two independent pathologists performed the histological examination and applied the scoring system in a blinded fashion.

### Quantitative analysis of hydroxyproline

The uterine scar tissue samples collected for Hyp content assay were trimmed into rectangular pieces, which were about 50 mg. The Hyp concentration was measured by a chemical colorimetric method using a commercial detection kit (A030 Hydroxyproline Detection Kit, Nanjing Jiancheng Bioengineering Institute, Nanjing, China).

### Immunohistochemistry

Sections of uterine scar from the rats were used to confirm and compare the expression levels of connective tissue growth factor (CTGF) and CD34. Paraffin-embedded tissue samples were deparaffinized in xylene and rehydrated in graded ethanol. Antigen recovery was performed in 10 mmol/L boiling sodium citrate buffer at pH 6.0 for 10 min at 92–98°C; then the specimens were incubated with 0.3% H_2_O_2_ for 15 min. Non-specific binding was blocked with normal horse serum for 20 min at room temperature. The sections were incubated with polyclonal rabbit anti-CTGF and anti-CD34 antibody (diluted 1:100, Abcam) at 4°C overnight. The sections were washed with phosphate-buffered saline and incubated with biotinylated secondary antibody for 30 min (diluted 1:1,000, Zhongshan Golden Bridge Inc., China). Sections were then treated with ABC solution at 37°C for 30 min and incubated with 3, 3-diaminobenzidine for 5 min. Counterstaining was carried out with Harris hematoxylin. Cells with brown staining to membranes or in the cytoplasm were considered positive. Immunohistochemical scoring in the sample was evaluated by both the percentage and intensity of positive cells as previously described.

### Statistical analysis

Statistical analysis was performed using SPSS 22.0. Measurement data were presented as mean ± standard deviation. For continuous variables, Kolmogorov–Smirnov tests of normality were used to evaluate the distributions. Data were analyzed using Student’s *t*-test or Mann–Whitney *U* test. The correlations between groups for categorical variables were examined using the *c*^2^ test or Fisher’s exact test. Statistical significance was considered *P* < 0.05.

## Results

The body weight, uterine weight, and food intake among the groups did not show any significant differences on days 5, 10, and 15 (Table 1). The time for abdominal skin wound healing in all groups ranged from 4 to 8 days. Statistically, the mean healing time for skin wounds in the MFP groups was dose dependent and was 1–2 days less than the control group regardless of the feeding time (Table 2, *P* < 0.01).

**Table 1:** Comparison of body weight, uterine weight, and food intake among the groups

	Day	Control	SD	Fenchu groups (mg/kg)					
				1.1	SD	2.2	SD	4.4	SD
Body weight (g)	5	255.71	22.76	255.00	16.54	253.14	17.37	243.86	13.85
	10	267.14	20.84	251.14	30.02	262.00	28.52	258.86	25.16
	15	272.71	18.84	257.00	7.16	262.00	27.36	258.29	23.14
Uterine weight (g)	5	0.55	0.15	0.47	0.09	0.51	0.12	0.62	0.13
	10	0.59	0.10	0.48	0.15	0.49	0.13	0.55	0.17
	15	0.45	0.03	0.56	0.21	0.53	0.19	0.49	0.16
Food intake (g/24 h)	5	132.29	15.70	128.00	12.18	136.71	12.51	126.29	11.46
	10	139.57	13.49	128.29	11.91	128.43	12.87	135.00	8.77
	15	136.14	13.97	131.43	15.04	129.43	12.79	131.29	12.70

Values are presented as mean ± SD, *n* = 7 for each group. SD, standard deviation.

There were no significant differences among groups on days 5, 10, and 15.

**Table 2:** Abdominal skin wound healing time (d) among the groups^a^

Control	SD	MFP groups (mg/kg)					
		1.1	SD	2.2	SD	4.4	SD
6.43	0.87	6.19	0.68	5.67*	1.02	5.71**	1.01

a There is no significant difference among days 5, 10, and 15 after surgery.

Values are presented as mean ± SD, *n* = 21 for each group. SD, standard deviation.

Compared with the control group:

* *P*= 0.013

** *P*= 0.018.

### MFPs accelerated collagen and smooth muscle proliferation and maturation process in wounds

**Fig. 1:**
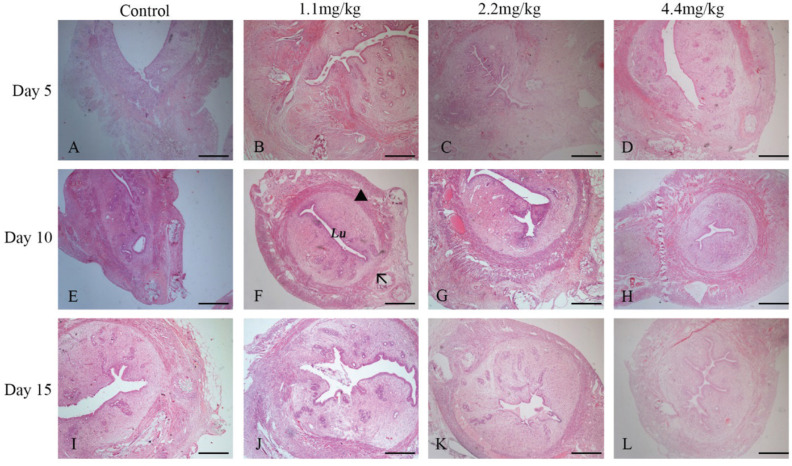
Representative histological images of hematoxylin–eosin staining at uterine surgery site were shown. The rats were treated with normal saline (the control group) or MFPs (1.1, 2.2, and 4.4 mg/kg, respectively) for 5, 10, and 15 days after surgery. The uterine wall of the normal rats has three layers. From innermost to outermost, these layers are endometrium, myometrium, and perimetrium. (F)**Lu** refers to the uterine lumen, **the black arrow** points to the uterine scar, and **the black triangle** marks the myometrium. Original magnification, ×100 (scale bar = 100 µm).

We identified the site of prior uterine CS surgery in the control group and 1.1, 2.2, and 4.4 mg/kg MFP groups at days 5, 10, and 15, respectively. Representative histological images of the uterine surgery site were shown in Fig. 1. H&E staining first showed that the uterine wall of the normal rats has three layers (Fig. 1). From innermost to outermost, these layers are endometrium, myometrium, and perimetrium. The endometrium consists of a single layer of ciliated columnar epithelium and the stroma on which it rests. The stroma is a layer of connective tissue and full of kinds of cells, which varies in thickness according to hormonal influences. Myometrium has two layers: outer longitudinal and inner circular fibers. Between them, there are loose connective tissues and blood vessels. The perimetrium is the serosa layer covering the outer surface of the uterus, which belongs to the visceral peritoneum.

Tissue integration was defined as apposition of the surgical wound edges. On day 5 after CS, wound adhesion was absent or slight in the control group, whereas all MFP rats had partial wound integration (Fig. 1A–D). On days 10 and 15, integration appeared complete in 2.2 and 4.4 mg/kg MFP groups (Fig. 1E–L). It seems that the healing process was influenced both by the days after surgery and MFPs intake. A dose-dependent and time-dependent character in the process of tissue integration was presented in the MFP groups.

**Fig. 2:**
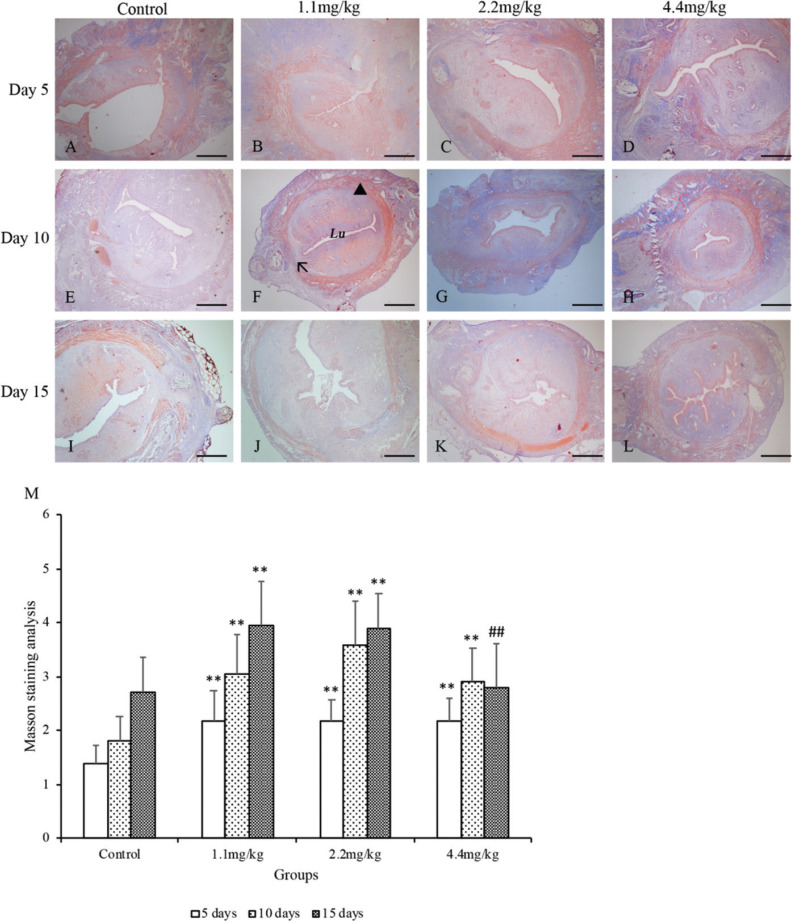
Masson’s trichrome staining of the uterine scar in rats. The rats were treated with normal saline (the control group) or MFPs (1.1, 2.2, and 4.4 mg/kg, respectively) for 5, 10, and 15 days after surgery. Representative histological images at the uterine surgery site were shown (A–L). The collagen was stained in blue, and the smooth muscle fiber was stained in red. (F) **Lu** refers to the uterine lumen, **the black arrow** points to the uterine scar, and **the black triangle** marks the myometrium. Original magnification, ×100 (scale bar = 100 µm). Morphological findings were semiqualitatively assessed under the light microscope using a scorning system including the percentage and intensity of positive cells (M). Values are presented as mean ± SD. Compared with the control group: *P < 0.05, **P < 0.01; compared with 1.1 mg/kg MFP group: #P < 0.05, ##P < 0.01.

The collagen deposition, fibroblast proliferation, and smooth muscle regeneration at the uterine surgery site were detected using Masson’s trichrome staining (Fig. 2). At the stage of day 5 after CS surgery (Fig. 2A–D), (Beijing Solarbio Science & Technology Co. Ltd., Beijing, China) collagen fibers in the MFP groups were present and better organized at the site of the uterine scar, compared with the control group (*P* < 0.01). The continuous smooth muscle fibers and layers similar to normal uterus were also found surrounding the myometrium in the MFP groups, whereas less organized, intermittent, or thin muscle bundles appeared in the control group. At the stage of days 10 and 15 after CS surgery, in the control group, blue staining could not identify any areas of mature collagen condensation (Fig. 2E–L), whereas, in the MFP groups, collagen was more prominent especially in the areas surrounding the surgical stitches (*P* < 0.01). Especially on day 15, the wound integration for MFP groups was complete, and the uterine architecture was restored to normal (Fig. 2L). The fibrinous exudate was absent, and there were no stitches visible. The circular muscle was restored around the intact lumen, and the serosa returned to normal as shown by the continuous external layer.

Conversely, in the control group, fibroblast proliferation at the surgical site was still visible and active (Fig. 2I). The site of the scar was recognized as a thinner tissue integration layer with discontinuous myometrium and serosa. Overall, these results suggest accelerated collagen and smooth muscle proliferation and maturation process in the MFP groups.

### MFPs enhanced skin wound tensile strength in rats

**Fig. 3:**
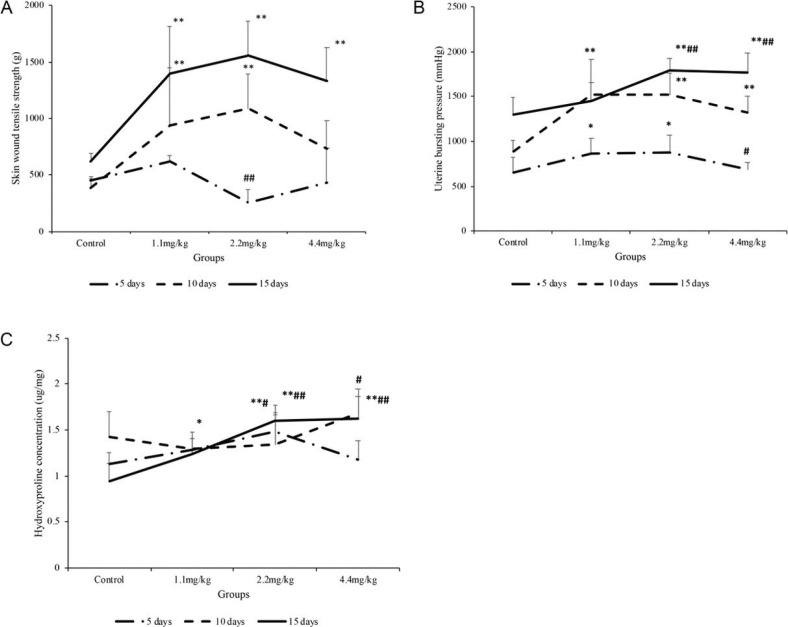
Analysis of skin wound tensile strength, uterine bursting pressure, and hydroxyproline levels at the uterine scar site. The rats were treated with normal saline (the control group) or MFPs (1.1, 2.2, and 4.4 mg/kg, respectively) for 5, 10, and 15 days after surgery. (A) Skin wound tensile strength measured on days 5, 10, and 15 from the control and MFP groups. (B) Uterine bursting pressure measured on days 5, 10, and 15 from the control and MFP groups. (C) Hydroxyproline levels at the uterine scar site measured on days 5, 10, and 15 from the control and MFP groups. Values are presented as mean ± SD. Compared with the control group: *P < 0.05, **P < 0.01; compared with 1.1 mg/kg MFP group: #P < 0.05, ##P < 0.01.

The skin wound tensile strength continued to increase from 5 to 15 days after CS surgery (Fig. 3A). Our results showed that the skin wound tensile strength of rats in 1.1 and 2.2 mg/kg MFP groups was significantly higher than those in the control group on days 10 and 15 postcesarean, respectively (*P* < 0.01), while the same increase was also found in the 4.4 mg/kg group on day 15 (*P* < 0.01).

### MFPs improved uterine bursting pressure in rats

Similarly, the uterine bursting pressure kept increasing from days 5 to 15 after CS surgery (Fig. 3B). The 1.1 mg/kg MFP group had greater bursting pressure with a significant increase than those in the control group on days 5 and 10 (*P* < 0.05), whereas the 2.2 mg/kg MFP group showed a significant trend to increase at all time points (*P* < 0.05). On the other hand, rats in the 4.4 mg/kg MFP group showed a higher bursting pressure on days 10 and 15 and reached its maximal stiffness on day 15 (*P* < 0.01). Moreover, MFPs intake with 2.2 and 4.4 mg/kg on day 15 was found to offer a significant improvement in the uterine bursting pressure compared with the 1.1 mg/kg MFP group (*P* < 0.01).

### MFPs promoted Hyp expression in wounds

As Hyp is the main component of collagen fibers in connective tissue, its level represents the amount of collagen and granulation tissue formation. In the uterus, the Hyp concentration increased significantly in the 1.1 mg/kg MFP group compared with the control group on day 15 (Fig. 3C, *P* < 0.05), while in the 2.2 mg/kg MFP group, the increase in Hyp concentration did show any significant difference on days 5 and 15 (*P* < 0.05). Rats in the 4.4 mg/kg MFP group were also found to be more Hyp expression on day 15 (*P* < 0.01). Furthermore, significant collagen deposition was observed in uterine wound tissue in the 2.2 and 4.4 mg/kg MFP groups compared with the 1.1 mg/kg MFP group at day 15 postcesarean (*P* < 0.01).

### MFPs increased CD34 and CTGF expressions in wounds

**Fig. 4:**
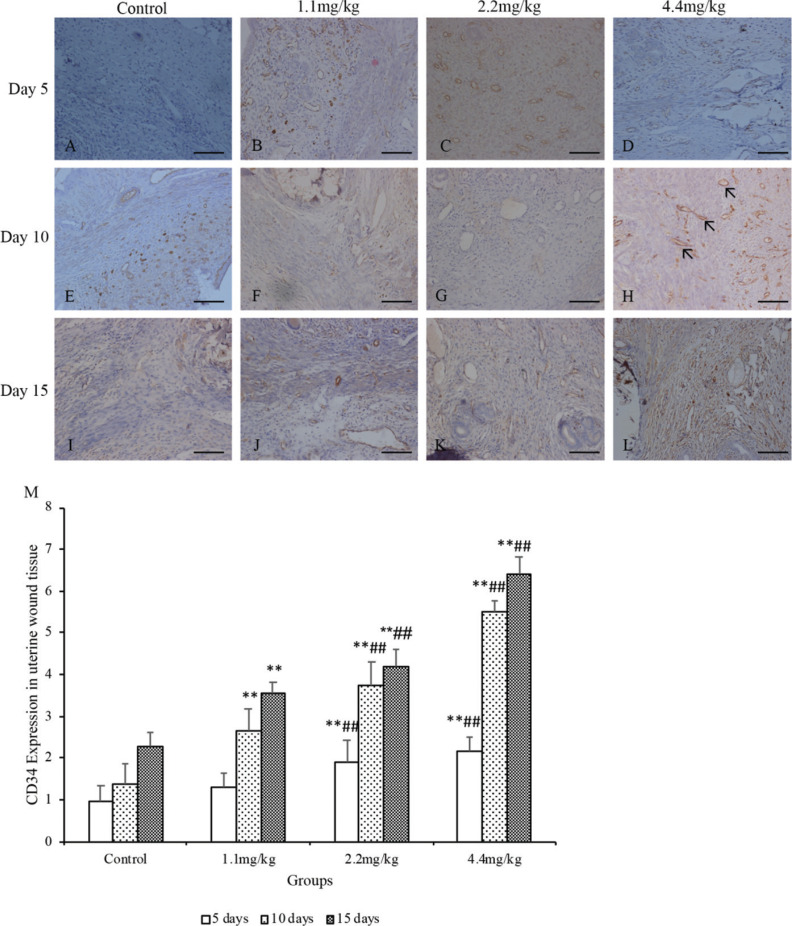
CD34 expression of the uterine scar in rats. The rats were treated with normal saline (the control group) or MFPs (1.1, 2.2, and 4.4 mg/kg, respectively) for 5, 10, and 15 days after surgery. Representative histological images at uterine wound sections were shown (A–L). Cells with brown staining to membranes were considered positive. (H) The **black arrows** point to the blood vessels in the uterine scar. Original magnification, 5×40 (scale bar = 100 µm). Morphological findings were semiqualitatively assessed under the light microscope using a scorning system including the percentage and intensity of positive cells (M). Values are presented as mean ± SD. Compared with the control group: *P < 0.05, **P < 0.01; compared with 1.1 mg/kg MFP group: ^#^*P* < 0.05, ^##^*P* < 0.01.

**Fig. 5:**
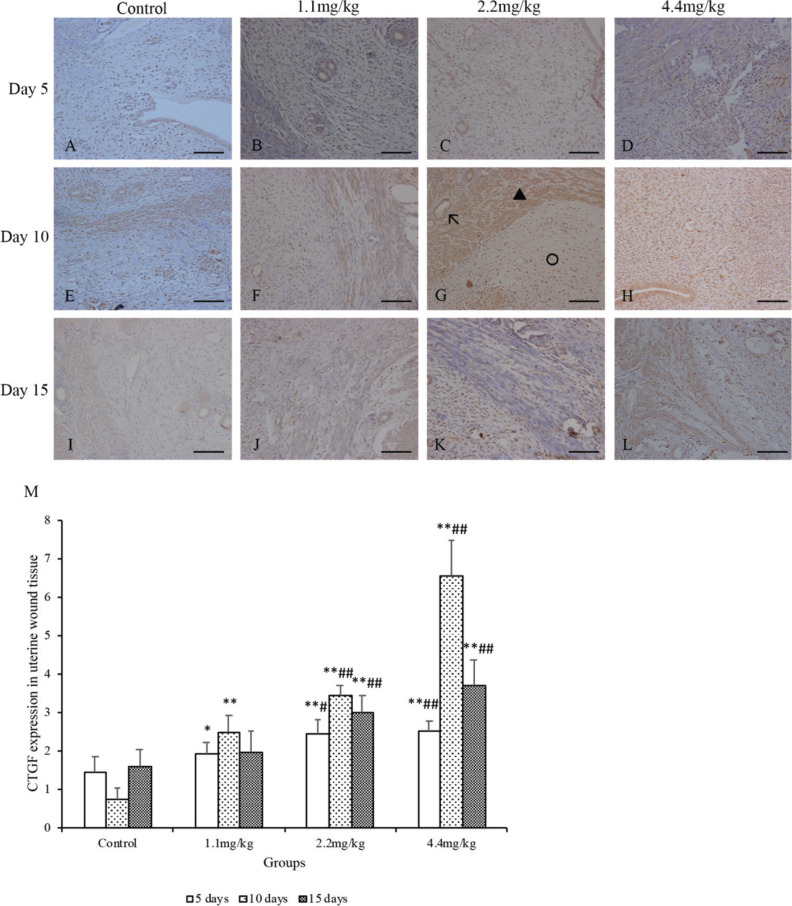
Connective tissue growth factor (CTGF) expression of the uterine scar in rats. The rats were treated with normal saline (the control group) or MFPs (1.1, 2.2, and 4.4 mg/kg, respectively) for 5, 10, and 15 days after surgery. Representative histological images at uterine wound sections were shown (A–L). Cells with brown staining to membranes or in the cytoplasm were considered positive. (G) The **black arrow** points to the blood vessel, the **black triangle** marks the myometrium, and the **black circle** refers to the stroma of endometrium. Original magnification, 5 × 40 (scale bar = 100 µm). Morphological findings were semiqualitatively assessed under the light microscope using a scorning system including the percentage and intensity of positive cells (M). Values are presented as mean ± SD. Compared with the control group: *P < 0.05, **P < 0.01; compared with 1.1 mg/kg MFP group: ^#^*P* < 0.05, ^##^*P* < 0.01.

The CD34 and CTGF immunoreactivity of uterine wound sections were examined in the MFP and control groups at 5, 10, and 15 days postcesarean. The number of cells expressing CD34 and CTGF were counted. As shown in Fig. 4, the CD34 expression was significantly higher in the 1.1 mg/kg MFP group than those in the control group at days 10 and 15 postcesarean (*P* < 0.01); the CTGF expression was also significantly higher at days 5 and 10 postcesarean (Fig. 5, *P* < 0.05). For rats in the 2.2 and 4.4 mg/kg MFP groups, both CD34 and CTGF expressions were significantly higher when compared with the control group at days 5, 10, and 15 postcesarean (*P *< 0.01). Besides 5, 10, and 15 days after CS surgery, significantly more CD34 and CTGF depositions were visualized at the wound site of the rats in the 2.2 and 4.4 mg/kg MFP groups compared with the 1.1 mg/kg MFP group (*P* < 0.01 and 0.05, respectively).

## Discussion

In the present study, we sought to determine whether dietary intake of MFPs affects the healing process of skin and uterine incision in rats following CS. Using the CS rat model, this study demonstrated that in the MFP groups, the skin tensile strength, uterine bursting pressure, and Hyp were significantly higher than those in the control group at all three time points. The formation of collagen and smooth muscle fibers and the expression of CD34 and CTGF at the incision site were increasingly observed in the MFP groups. These results indicated that MFPs have a great potential to accelerate the process and quality of wound healing in rats after CS.

A direct comparison in wound healing between humans and animals is difficult. Prior animal studies demonstrated that the period of gestation for the rat is 20 days, whereas for the human is 40 weeks (13, 34). It means that each day of gestation for the rat is approximately equivalent to 2 weeks of gestation for the human. Therefore, we speculated that these intervals, days 5, 10, and 15 after CS, may have a significant clinical relevance to humans (13), which reflect the stages of inflammation, proliferation, and maturation on uterine wound healing in rats. However, the time points choosing for study could vary with different animal models (phenotypical differences) or study design (13, 35).

Nutrition definitely plays an essential role in the wound healing process (23). The major physiological changes, such as collagen synthesis, angiogenesis, and wound maturation, need both energy and nutritional substrates (36). Exogenous collagen peptides intake could enhance protein synthesis and anabolic effects, which have been demonstrated significant beneficial effects in rats (35, 36). Since MFPs are food-derived peptides that do not contain any pharmaceutical ingredients, the oral safety can be promised. Moreover, the distinct increase in protein synthesis might be a potential effect of MFPs intake. The body needs a higher amino acid intake to guarantee the collagen production in the hypermetabolic state, such as postpartum or postsurgery period (36), yet the oligopeptides are believed to be absorbed more easily than macromolecular proteins (37). Wang et al. (35) found that marine collagen peptides, enzymatically hydrolyzed from the skin of chum salmon, were able to promote wound healing in CS or diabetic rats. Whey peptides (WPs), a kind of bioactive peptides derived from whey proteins, had a significant wound healing potential in the skin incision or the uterus (36).

The tensile strength or bursting pressure measurement has been used extensively, is a common parameter in the evaluation of wound healing, and serves as a useful indicator for predicting the performance of an incision under clinical conditions of physical stress (29). As the tensile strength of the scarred tissue is much weaker than that of the normal tissue, a higher tensile strength reflects a faster or healthier healing process at the wound site. In this study, we have observed significantly accelerated stability, stiffness, and elasticity with dose dependently and time dependently in the MFP groups compared with the control group. The higher bursting pressures found in the postpartum experiment probably reflect the thicker uterine walls that rich in collagen content and organized smooth muscle fibers. Therefore, we assumed that the healing aspects of the uterine incision can mostly influence the risk of uterine rupture. Bowel et al. (29) found that the uterine horn bursting pressure in the experimental animals was significantly greater than those in the control group for both 28 days postpartum and near term in a subsequent pregnancy. However, whether our observed increase in incisional strength after MFPs treatment means that the uterine scar will be less likely to rupture during labor is yet uncertain.

Histological examination in the present study demonstrated significant differences in scar integration, level of angiogenesis, collagen synthesis, smooth muscle proliferation, and arrangement at the site of myometrial incision between the MFP and the control groups. MFPs were found to greatly help the uterine wound architecture restored to normal. The process could be concluded that, first, significant collagen deposition was observed at the site of incision. The collagen fibers then were arranged neatly, and a small number of smooth muscle cells were seen extending from the periphery. Over time, the smooth muscle tissue gradually increased, and the scar area of the incision was mainly covered by smooth muscle tissue and collagen fibers. Conversely, hyperplastic fibrous connective tissue means vulnerable and inelastic structure and risk of instability. As a result, collagen is required, is essential for wound healing, which involved in the initial phase of myometrial wound healing, and may be responsible for the observed differences in the stiffness and elasticity of the uterine scar. Some studies also indicated that the skin tensile strength or uterine bursting pressure increases rapidly as collagen deposition increases and cross-linkages formed between the collagen fibers (35, 36).

MFPs are rich in Hyp according to the composition analysis (Supplementary materials). Hyp, derived from proline, is the main component of collagen fibers in connective tissue, and it is crucial for collagen biosynthesis, structure, and strength. Dietary proline is necessary for would healing in both animals and humans (38). Hyp content, as a marker of collagen deposition, always has a great positive influence on the tensile strength of connective tissue. At the same time, Hyp plays an important role in the formation and stability of triple helix of collagen molecules at a physiological temperature (39). Similar results have been obtained in the present study and also in previous studies (12, 29). By measuring the content of Hyp in the scar tissue of rat uterine incision, we can indirectly understand the effect of MFPs on the formation of collagen fibers in the uterine incision. Increasing Hyp content is beneficial to collagen synthesis, the stability of collagen molecules, tissue repair, and tensile strength. However, some studies reached different conclusions regarding the meaning of collagen content in uterine tissue. Wang et al. (35, 36) found that Hyp concentration changed differently in the skin and uterus, which meant WP or marine collagen peptides could increase the Hyp content in the skin incision, but not that in the uterine incision.

Vascular repair and angiogenesis are the most important factors for tissue repair and regeneration (40). Angiogenesis, which refers to the development of new blood vessels, allows transfer of oxygen, nutrients, and inflammatory cells to the site of a wound after injury, as well as contributes to granulation tissue formation and ultimately to wound integration (35). Previous studies have shown that CD34 can promote the migration of vascular endothelial cells under the combined action of adhesion molecules and chemokines, which are conducive to endothelial repair and vascular reconstruction (41–43). The expression of CD34, as a marker in tissue, can show the density of capillaries in the uterine scar tissue and reflect the healing process. In the present study, results suggested that MFP’s feeding could significantly contribute to the expression of CD34 and the proliferation of local neocapillaries in the uterine scar tissue. Thus, increased vascularization by MFPs at the uterine scar could greatly promote wound repair and healing, yet the mechanism needs to be further studied.

Wound healing is a dynamic and systematic process based on various mediators. CTGF is a growth factor that stimulates fibroblast proliferation and collagen deposition (44). It can be synthesized and secreted by fibroblasts, smooth muscle cells, and endothelial cells. CTGF is also a downstream factor for the tissue growth factor-beta to exert biological effects. CTGF has a strong regulatory effect on the differentiation and proliferation of fibroblasts and the synthesis and degradation of extracellular matrix (45). CTGF has important roles in many biological processes, including cell adhesion, migration, proliferation, angiogenesis, skeletal development, and tissue wound repair (44). In the present study, results further indicated that MFPs feeding could promote the synthesis and deposition of collagen, the differentiation and proliferation of fibroblasts for wound repair, by significantly enhancing the expression of CTGF around uterine scar. However, the regulatory mechanism by MFPs is not clear and needs to be further studied.

## Conclusions

The present study mainly indicated that medium and high doses of MFPs feeding after CS in rats could significantly promote the healing of rat uterine incision. Its promoting effect may be mainly related to the mechanism of promoting the formation of new capillaries in uterine scar tissue, the growth, and repair of collagen fibers and smooth muscle tissues. One of the limitations of this study is the lack of positive control as the effective medical intervention methods are not yet available. On the other hand, compared to the rat uterus, the human uterus has a different anatomical structure and physiological process, so further study should be conducted on human subjects, and more studies are needed to ensure the functions and thoroughly explore the mechanisms of MFPs in the wound healing process.

## Supplementary Material

Marine fish peptides (collagen peptides) compound intake promotes wound healing in rats after cesarean section
