# Supplementary material for: Marine fish peptides (collagen peptides) compound intake promotes wound healing in rats after cesarean section
Source: Food Nutr Res. 2020 Aug 31;64:10.29219/fnr.v64.4247. doi: 10.29219/fnr.v64.4247 (PMC7534952; doi:10.29219/fnr.v64.4247)
Supplement: Marine fish peptides (collagen peptides) compound intake promotes wound healing in rats after cesarean section [file FNR-64-4247-s001.doc]

**Preparation and identification of the main ingredients of MFPs**

1. **Freshwater fish peptides**

Freshwater fish peptides were offered by Vinh Hoan Collagen Cooperation. The perapation process was as follows: firstly, the gelatin was extracted from hypophthalmus fish skin. Secondly, the solution was spry dried to finished products after proper enzymatic hydrolysis. The nutrient composition is as follows:

**Table 1**. Composition of amino acid of Freshwater fish peptides (g/100g)

| Composition | Content |
| --- | --- |
| Ala | 9.5 |
| Arg | 0 |
| Asp | 4.5 |
| Cys | 0 |
| Glu | 7.2 |
| Gly | 23.9 |
| His | 0.5 |
| Ile | 1.2 |
| Leu | 2.5 |
| Lys | 2.9 |
| Met | 1.2 |
| Phe | 1.9 |
| Pro | 12.3 |
| Ser | 3.3 |
| Thr | 2.4 |
| Trp | 0 |
| Tyr | 0.3 |
| Val | 2.5 |
| Hyp | 10.9 |

1. **Oligopeptides powder of marine fish**

The oligopeptides powder of marine fish was made from marine fish skin supplied by Zhejiang Haishi Biology Technology Company. The perapation process was as follows: firstly, fresh marine fish skin was grounded up, mixed with water and emulsified. Secondly, the bacillus licheniformis protease (0.15%) and bacillus subtilis protease (0.1%) were added to the mixture and enzymatic hydrolysis was conducted for 3 hours. Enzyme was instantaneously killed at high temperature. Finally, the mixture anter after enzymatic hydrolysis was filtered, desalted, concentrated, decolored and spray dried to obtain the end-product.

**Table 2**. Chemical index of Oligopeptides powder of marine fish

| Index | Content |
| --- | --- |
| Protein | 88% |
| average molecular weight | 400-600 |

1. **Taihe silk chicken enzymatic hydrolysate**

Taihe silk chicken enzymatic hydrolysate was made from Taihe silk chicken supplied by our own company. The perapation process was as follows: firstly, Taihe black chicken with removal of head, hair, claw and viscera was washed cleanly and processed into mincemeat by meat mincer (after proper pretreatment). Secondly, the mincemeat was enzymatic hydrolyzed for 4 hours at 55℃with papain (2% ) and then fixed volume after refined. The nutrient composition is as follows detected by automatic amino-acid analyzer:

**Table 3**. Composition of amino acid of Taihe silk chicken enzymatic hydrolysate (g/100g)

| Composition | Content |
| --- | --- |
| Ala | 6.1 |
| Arg | 4.9 |
| Asp | 6.2 |
| Cys | 0.7 |
| Glu | 11.6 |
| Gly | 11.0 |
| His | 2.8 |
| Ile | 2.3 |
| Leu | 4.6 |
| Lys | 5.0 |
| Met | 1.4 |
| Phe | 2.4 |
| Pro | 5.8 |
| Ser | 3.0 |
| Thr | 2.8 |
| Trp | 1.0 |
| Tyr | 1.5 |
| Val | 3.7 |

1. **Whey protein hydrolysate**

Whey protein hydrolysate was made from whey protein supplied by Arla. The perapation process was as follows: whey protein was mixed or homogenized in water, processed by heat treatment, enzymatic hydrolyzed, processed again by heat treatment and spray dried into end-products after filtered. The chemical index of whey protein hydrolysate is followed（offered by manufacturer):

**Table 4**. Chemical index of whey protein hydrolysate

| Composition | Content |
| --- | --- |
| Protein | 83% |
| Lactose | ≤4% |
| average molecular weight | 750-1100 |
| molecular weight | 450-700 |

1. **Water-soluble dietary fiber**

Water-soluble dietary fiber was made from corn starch supplied by Songgu Chemical Industry Co., Ltd. The perapation process was as follows: corn starch was obtained by spray drying after heat treatment, acid treatment and enzyme treatment. Nutrient composition of water-soluble dietary fiber is as follows:

**Table 5**. Chemical index of whey protein hydrolysate

| Composition | Content |
| --- | --- |
| Water-soluble dietary fiber | ≥90% |
| Lactose | 2% |

**6. Casein phosphopeptide**

Casein phosphopeptide was made from casein supplied by Hangzhou Kangyuan Food Technology Company. The perapation process was as follows: casein after enzymatic hydrolysis was spray dried to obtain the end-product. Nutrient composition of casein phosphopeptide is as follows:

**Table 6**. Nutrient composition of casein phosphopeptide

| Composition | Content |
| --- | --- |
| Casein phosphopeptide | 17.56% |
| Protein | 80.64% |

**7. Zinc gluconate**

Gluconolactone was used as the starting material and reacted with zinc compound to obtain the end-product, which was supplied by Zhengzhou Purui Biological Engineering co., LTD. Nutrient composition of Zinc gluconate is as follows:

**Table 7**. Nutrient composition of Zinc gluconate

| Composition | Content |
| --- | --- |
| Zinc gluconate | 99.15% |
| Dry weight loss | 7.36% |
